# Hematopoietic and Chronic Myeloid Leukemia Stem Cells: Multi-Stability versus Lineage Restriction

**DOI:** 10.3390/ijms232113570

**Published:** 2022-11-05

**Authors:** Geoffrey Brown

**Affiliations:** Institute of Clinical Sciences, School of Biomedical Sciences, College of Medical and Dental Sciences, University of Birmingham, Birmingham B15 2TT, UK; g.brown@bham.ac.uk; Tel.: +44-(0)12-1414-4082

**Keywords:** chronic myeloid leukemia, leukemia stem cells, hematopoietic stem cells, oncogenes, epigenetics

## Abstract

There is compelling evidence to support the view that the cell-of-origin for chronic myeloid leukemia is a hematopoietic stem cell. Unlike normal hematopoietic stem cells, the progeny of the leukemia stem cells are predominantly neutrophils during the disease chronic phase and there is a mild anemia. The hallmark oncogene for chronic myeloid leukemia is the *BCR-ABLp210* fusion gene. Various studies have excluded a role for *BCR-ABLp210* expression in maintaining the population of leukemia stem cells. Studies of *BCR-ABLp210* expression in embryonal stem cells that were differentiated into hematopoietic stem cells and of the expression in transgenic mice have revealed that BCR-ABLp210 is able to veer hematopoietic stem and progenitor cells towards a myeloid fate. For the transgenic mice, global changes to the epigenetic landscape were observed. In chronic myeloid leukemia, the ability of the leukemia stem cells to choose from the many fates that are available to normal hematopoietic stem cells appears to be deregulated by BCR-ABLp210 and changes to the epigenome are also important. Even so, we still do not have a precise picture as to why neutrophils are abundantly produced in chronic myeloid leukemia.

## 1. Introduction

From findings in the 1950s, tumorigenesis is a multistep process [1] and a complex sequence of events needs to be completed to transform a normal cell into a cancerous one. The identification of oncogenes has been one of the major advances to unraveling the biology of cancer. Oncogenic insults to a cell arise from cellular genes (proto-oncogenes) that are mutated, and then typically dominant in nature, and chromosomal translocations, that give rise to a constitutively active fusion protein, such as a signaling kinase, or that place a strong promoter close to the involved genes leading to overexpression. The normal gene products control cell survival, proliferation or differentiation, and the oncogene products essentially deregulate the controls on these processes. Hence, oncogenes have an intrinsic capacity to control cell survival, proliferation, and differentiation.

However, the induced expression of an oncogene within cells, even to a very high level, does not lead invariably to cell transformation. The *BCR-ABLp210* fusion gene (also termed *BCR-ABL1*) arises from the t(9; 22) (q34; q11) reciprocal translocation, is characteristic of chronic myeloid leukemia (CML), and the chimeric protein has constitutive kinase activity. Expression of the BCR-ABLp210 protein at a high level did not transform NIH-3T3 cells, but expression of BCR-ABLp210 with C terminal rearrangements transformed permissive NIH-3T3 subclones [2,3]. Changes to the C terminal had activated the transformation capacity of BCR-ABLp210, and a particular function domain(s) is/are required for transformation. The transformation of particular subclones brings to attention that there is an important dynamic relationship between the functionality of the BCR-ABLp210 protein and constraints that prevail within the intracellular environment. Cell status also influences the precise nature of the outcome from oncogene expression within cells. The oncogenes *CTNNB1*, *TERT*, and *MYC* are characteristic of liver cancer, but they induced senescence in primary human hepatocytes and fibroblasts, and when fibroblasts were reprogrammed to a liver progenitor cell (induced hepatocytes) they transformed these cells [4]. The environment a cell resides in is a further consideration to whether an oncogene transforms a cell or fails to do so. Mutant KRAS-G12V induces pancreatic ductal adenocarcinoma in mice when expressed in embryonic acinar lineage cells whereas chronic pancreatitis is needed for KRAS-G12V to induce pancreatic tumors in adult mice [5]. For adult mice, changes to the environment will have altered the intracellular status of cells. Barr has argued in favor of cell context-specific mechanisms of transformation because there is also the need for the avoidance of any oncogene-mediated toxic effects and a complex relationship exists between a translocation event and the juxtaposed loci [6]. The oncogene’s nature, the target cell’s status, and the environment a cell resides in are all part of the complex series of events for cancer.

As target cell status is important to successful transformation, we need to be certain about the cell-of-origin of a cancer to unravel its transformation process. This review focuses on CML because it is well known to arise from a hematopoietic stem cell (HSC), and, as mentioned above, its hallmark oncogene *BCR-ABLp210* is well established. The course of the disease is complex. Neutrophils are massively over produced at disease presentation and during the chronic phase. At three to five years after onset, CML generally progresses to a blast and accelerated phase. Two thirds of cases have blasts with a phenotype similar to that of acute myeloid leukemia (AML) cells. The remaining cases have blasts with a lymphoid morphology. Consideration is given to the influence of *BCR-ABLp210* expression on the behavior of CML leukemia stem cells (LSCs) that give rise to the chronic phase of disease, and why the behavior of these cells is very different to that of HSCs.

## 2. CML Arises in an HSC

CML is a clonal disease arising from a pluripotent HSC, as revealed by studies in the late 1960s of the expression of the glucose-6-phosphate dehydrogenase (G-6-PD) isoenzymes A and B within cells from three women with CML [7,8]. For normal cells, only one *G-6-PD* gene is active in each female cell, due to X chromosome inactivation, and they are a mixture of cells that express either the A or B isoenzyme. One enzyme isotype is present in the blood granulocytes, erythrocytes, platelets, monocytes, macrophages, and B lymphocytes of CML patients’ cells. A stem cell that is common to the development of the above cell types is the origin of CML.

We can exclude a committed myeloid progenitor cell as the target for transformation in CML from consideration of the action of induced *BCR-ABLp210* expression. LSCs must self-renew to sustain leukemia. Common myeloid progenitors (CMP) and granulocyte/monocyte progenitors (GMP) do not self-renew and the BCR-ABLp210 protein lacks the capacity to confer self-renewal upon CMPs and GMPs. CMPs and GMPs were transduced with *BCR-ABLp210* and did not serially re-plate when cultured in methycellulose supplemented with cytokines, and *BCR-ABLp210* expression did not affect their survival and differentiation. When *BCR-ABLp210* transduced CMPs and GMPs were injected into a lethally-irradiated congenic mouse, there was no evidence of leukemia at the time of sacrifice between 113 and 240 days. In essence, *BCR-ABLp210* had not conferred an essential attribute of LSCs within committed murine myeloid progenitors [9].

An HSC origin for CML is not exceptional because some of the sub-types of AML arise from an HSC. Just a small fraction (0.01%) of human AML cells was able to initiate leukemia in nonobese diabetic-severe combined immunodeficient mice, their cell surface phenotype corresponded to that of a normal HSC (CD34++, CD38-), and, like HSCs, the cells that initiated leukemia were able to self-renew. These findings led to the cancer stem cell model which states that rare cancer stem cells (CSCs) generate the hierarchy of cells that sustains a cancer, with the progeny of CSCs differentiating either partially or fully [10,11].

A general view is that leukemias and other cancers, originate from a rare population of cells. From early mapping of the various hematopoietic progenitor cells (HPC), these rare cells were included as ‘target’ cells for transformation for some of the human leukemias [12]. Acute promyelocytic leukemia (AML-M3) was seen as arising in a myeloid-committed progenitor, as there is an excessive production of promyelocytic blasts [13,14]. Childhood acute B-cell lymphoblastic leukemia (B-ALL) and T-cell acute lymphoblastic leukemia were described as arising in a B-cell lineage and T-cell lineage committed progenitors, respectively, as there is an excessive production of B-cell lineage blast cells and T-cell lineage blast cells, respectively [12]. More recent analyses have revealed a more primitive origin for acute promyelocytic leukemia [15], childhood B-ALL [16,17,18], and infant *MLL-AT4* B-cell precursor ALL [19]. Chronic lymphocytic leukemia has long been seen as arising in an antigen-experienced B-cell, whereas new evidence supports the view that this leukemia arises from transformation of an HSC [20,21]. The origins of some leukemias, for example, childhood B-ALL, is still a matter of debate [22]. Even so, it is conceivable that all of the leukemias arise from an HSC, with cancers in general arising from a tissue-specific stem cell. CSCs have been identified for bladder [23], head and neck squamous cell carcinoma [24], lung [25], pancreatic [26], prostate [27], and sarcoma [28], but there is often uncertainty regarding whether their normal counterpart is a tissue-specific stem cell or a progenitor cell because of a lack of appropriate markers.

## 3. CML LSCS Are Restricted to Neutrophil Production during Chronic Phase

The most striking feature of CML LSCs is a massive over production of neutrophils during the chronic phase of disease, and there is a mild anemia. This restriction of CML LSCs to myeloid cell production during the chronic phase is supported by findings from the induced expression of *BCR-ABLp210* in transgenic mice. The targeting of *BCR-ABLp210* to the bone marrow hematopoietic stem and progenitor cells, via stem cell antigen (*Sca1)-BCR-ABLp210*, led to a leukemia resembling human chronic phase CML [29,30]. *BCR-ABLp210* expression was also targeted to the bone marrow stem cell compartment using the tetracyclin (tet)-off system. The transgenic mice developed a human CML-like disease upon the induction of *BCR-ABLp210* expression (tet withdrawal), and the disease was transplantable by the use of bone marrow cells that lacked lineage markers (lin-) and that expressed the Sca-1 antigen and the c-kit receptor (c-kit+) for stem cell factor (termed LSK), which is a classical signature for HSCs. The disease was fatal in the transgenics and primary transplant recipients [31]. From these findings, the *BCR-ABLp210* oncogene is able to restrict very primitive bone marrow cells to a neutrophil fate. Perhaps cell status-related factors, e.g., transcription factors and cell signaling, interact with BCR-ABLp210 leading to a massive expansion of myeloid cells.

## 4. What Is the Role of BCR-ABLp210?

For many years, CML was seen as a “one-hit tumor”, and therapeutic approaches focused on achieving complete inhibition of the kinase activity of the BCR-ABLp210 protein. However, the kinase activity is not required for the survival and self-renewal of CML LSCs because they are insensitive to the second-generation inhibitors dasatinib, nilotinib, and bosutinib that are used to treat patients, and their persistence leads to minimal residual disease in patients [32,33]. For human CD34+ CML cells, BCR-ABLp210 knockdown achieves partial inhibition of BCR-ABLp210 activity and the addition of dasatinib completely inhibited the phosphorylation of CrkL and STAT5. A substantial proportion of the CD34+ CML cells survived (~50%) when cultured [31]. For the tet-off *BCR-ABLp210* transgenic model and when BCR-ABLp210 expression was shut off, CML LSCs persisted in vivo and upon re-expression of BCR-ABLp210 they were able to initiate leukemia in secondary recipients [31].

We might exclude BCR-ABLp210 from having a role in the maintenance/proliferation of CML LSCs, and therefore does induced *BCR-ABLp210* expression influence their differentiation? The engineered expression of *BCR-ABLp210* in embryonal stem (ES) cells allowed sustained expression when these cells were differentiated by using OP9 cell layers to provide support. The BCR-ABLp210+ ES cells differentiated into hemangioblasts which produced HSCs, HPCs, and finally mature blood cells. The outcomes from the expression of the *BCR-ABLp210* gene were two-fold. Multipotent and myeloid HPCs were increased and there was suppression of erythroid progenitor cell development. From in vitro colony formation assays, there was a dominance of myeloid colonies, with the balance of myeloid to erythroid colonies shifting from 1:2 to 4:1. Therefore, *BCR-ABLp210* expression can directly and acutely expand a very immature cell type with multilineage differentiation capabilities (c-kit+, lin- or CD34+, lin- cells) and change the balance of lineage development to favor myeloid development, despite the presence of erythropoietin (Epo) in cultures. Tet-regulated expression of BCR-ABLp210 allowed investigation of whether the change to the balance of erythroid versus myeloid colonies was reversable. Shutting of *BCR-ABLp210* during the last phase of culture development led to a normal erythroid over myeloid dominance [34].

Though BCR-ABLp210 can direct the lineage fate of HSCs, the presence of the BCR-ABLp210 protein alone may not be sufficient to initiate the chronic phase of human CML. BCR-ABLp210 mRNA, as encoded by the Philadelphia chromosome 22 (chromosome 22 with a piece of 9 attached), is present at a very low level in the cells of individuals who do not succumb to CML [35]. In earlier studies, investigators followed myelodysplasia patients who eventually developed CML by using X-chromosome linked G-6-PD A or B isotype expression and observed a peripheral clonal dominance prior to the development of CML. [36]. As mentioned above, CML clones, as identified by G-6-PD isotype expression, can differentiate into mature blood granulocytes, erythrocytes, platelets, monocytes/ macrophages, and B lymphocytes. The pathogenesis of CML may be, at least, a two-step process. Another possibility to explain the above phenomenon is that the translocation might be present in a more differentiated cell that is resistant to the oncogenic effect of the fusion protein, instead of being in the right, HSC, target cell.

## 5. Is Another Event Needed for CML before or after the Philadelphia Chromosome?

How might other genetic and epigenetic events, facilitated by BCR–ABLp210 or otherwise, lead to the onset of the chronic phase of CML? Cells that express BCR–ABLp210 accumulate genetic abnormalities, and this led to the proposal that BCR-ABLp210 is a multifaceted promotor of DNA mutation [37]. Possibilities are that the expression of BCR-ABLp210 leads to error cascades, and an accumulation of errors by influencing DNA repair or making changes to the accumulation of DNA damage. Regarding DNA repair, the BCR-ABLp210 tyrosine kinase facilitates the repair of DNA double-strand breaks, and other investigators postulated that genetic instability within CML cells may be due to unfaithful repair of double stranded breaks [38]. BCR-ABLp210 is known to enhance the DNA damage that is provoked by endogenous reactive oxygen species and exogenous genotoxic agents (reviewed in [39]). Presently, the precise involvement of BCR-ABLp210 in DNA repair processes is unclear.

There is good evidence to support the view that alterations to the epigenome plays a role in the development of the chronic phase of CML. The tet-inducible transgenic model of CML was used to show that BCR-ABLp210 triggers DNA methylation changes. DNA methylation patterns were examined for cells that were harvested as HSCs from non-induced and control *BCR-ABLp210* mice, induced and leukemic *BCR-ABLp210* mice, and repressed and rescued *BCR-ABLp210* mice. Cells from the leukemic mice showed a moderate increase of CpG islands’ DNA methylation levels. The investigators argued that a single oncogenic protein can trigger changes to DNA methylation at several gene loci and that the epigenetic abnormalities lead to the escape of the leukemic clone resulting in disease [40]. A pathway to widespread DNA methylation changes involves a class of RNAs called DNA (cytosine-5)-methytransferase 1 (DNMT1)-interacting RNAs. They originate from transcriptionally active gene loci and bind with high affinity to DNMT1 to prevent DNA methylation of the corresponding gene loci. BCR-ABLp210 might negatively regulate the expression of DNMT1-interacting RNAs to cause the silencing of specific genes [41]. From analysis of the proportions of methylated and unmethylated genes in CML stages, other investigators proposed that DNA methylation is increased in advanced disease and is associated with disease progression, resistance to imatinib, and shortened survival [42].

A significant loss of methylation at CpG islands that have a low-to-moderate level of methylation in wild-type HSPCs was reported for cells harvested as HSCs/HPCs from the *Sca1-BCR-ABLp210* transgenic mice. This change was lasting within the mature leukemic myeloid cells, despite an absence of oncogene expression in the mature leukemic cells. DNMT1 was upregulated within HSCs/HPCs from the mice, and the expression of *Dnmt1* in HSCs/HPCs, under control of the *Sca1*, led to malignancies that were mostly myeloid with a marked expansion of granulocytes in the bone marrow and blood. DNA hypomethylation was observed for cells from *Sca1-DNMT1* transgenic mice, and the pattern was similar to that observed for the cells from the *Sca1-BCR-ABLp210* mice. The investigators concluded that slight perturbations to the function of DNMTs are sufficient for the chronic phase of CML because epigenetic reprogramming by itself was sufficient to drive leukemogenesis. Again, global changes are important and DNMT1, DNMT3A, and DNMT3B interact with EZH2, the catalytic subunit of PRC2. Overexpression of DNMT1 may have sterically hindered the association between DNMT3A and EZH2, and DNMT3A homo-tetramers efficiently methylate cytosine leading to global hypomethylation [30].

Perturbation to the chromatin landscape has been shown for mutant RUNX1 oncoproteins that are able to guide HSC development. Expression of four types of RUNX1 was induced in ES cells which were then differentiated towards hematopoietic cells. RUNX1-ETO expression led to a bias towards a B cell identity by reducing the expression and binding of transcription factors (TF) that regulate myeloid differentiation (PU.1 and C/EBPα). Expression of R201Q, which has a mutation in the DNA binding domain, led to a bias away from megakaryocyte differentiation by reducing the interaction of wild-type RUNX1 with CBFβ (a master regulator of hematopoiesis) and increasing GATA1 binding (a TF for erythropoiesis). Differentiation of myeloid and erythroid cells was reduced by RUNX1-EV11 expression. Expression of the mutant proteins perturbed chromatin priming of lineage-specific sites [43], and RUNX1 plays a role in the organization of the chromatin landscape at the onset of hematopoiesis in order to maintain accessibility [44,45], and shapes landscapes via a cascade of direct and indirect targets [46].

Changes to the epigenetic landscape, which in turn control TF and signaling pathway components, are required for LSCs to initiate the chronic phase of CML LSCs, and, in 1957, Waddington proposed that an epigenetic landscape dictates stem-cell decision-making. Developing stem and progenitor cells roll down valleys that branch towards an end-fate with the ridges to the hills maintaining a chosen fate [47]. The valleys and hills are the epigenetic landscape that controls the expression of key transcription factors. The existing landscape has also been described as “the judge, jury, and executioner of stem cell fate” [48]. From the ability of BCR-ABLp210 to increase myeloid progenitor cell development and suppress erythroid progenitor cell development by HSCs (derived from hemangioblasts), BCR-ABLp210, either alone or in combination with other factors, can mis-shape the epigenetic landscape.

## 6. HSC Decision-Making for Lineage Fate

An understanding of how HSCs choose a particular pathway of development is clearly important to unraveling the lineage restriction of CML LSCs. Therefore, what is the underlying principle to HSC decision-making? A general view is that cells make binary decisions that are irreversible such as all-or-nothing decisions. The cellular controls are bi-stable, with cells switching from one steady state to another when there is a change to external and/or internal systems. Well-studied examples are whether to undergo apoptosis or not and to mature or not, with both based on information from the environment reaching a threshold level which is processed to an outcome [49]. Binary decision-making, as depicted as a tree-like process, also underpins models for the development of the different cells of an entire organism and longstanding fate maps for hematopoiesis. In classic models, HSCs first choose between the myeloid and lymphoid pathways of development. A series of stepwise decisions then progressively restrict lineage options to ultimately give rise to single lineage-restricted HPCs. Routes to each cell type are via preferred intermediate HPCs [50].

For HSCs and to add to the choice of a developmental pathway, there is the need to integrate controls on various other cell states. They include the maintenance of survival and choices between quiescence versus cell division and self-renewal versus differentiation. Multi-stability allows cells to process more information and to regulate gene expression for more than two mutually exclusive stable states. It exists for gene regulatory networks [51,52], signaling pathways [53,54], and metabolic networks [55]. Multi-stability would allow HSCs to switch to an appropriate state to accord with the various changes to external influences, and modelling has revealed that it is important to HSCs choosing a cell lineage [56]. The TFs GATA1, GATA2, and PU-1 play essential roles in HSC and HPC development. GATA2 is a driver of hematopoiesis; a high level of expression of GATA1 is likely to veer HSPCs towards megakaryocyte/erythroid development, and a high level of expression of PU-1 veer HSPCs towards granulocyte/macrophage development. The framework to the mathematical modeling of the three TFs was based on the embedding of sub-systems with less stable states and the use of equations that took-into-account the genes in the system, their regulation, and the various equilibria. Two bi-stable models were embedded to achieve a tri-stable model which accorded with experimental data. The tri-stable model was then modified to achieve four stable states. For GATA1 and GATA-2, the modelling assumed that there is an exchange of GATA1 for GATA2 at the chromatin site, which then controls GATA1 and GATA2 gene expression. The simulated four stable states were maintenance of the HSC state related to unsuccessful GATA2 to GATA1 switching when the displacement of GATA2 is not sufficient, a myeloid progenitor state related to unsuccessful GATA-2 to GATA1 switching when the displacement of GATA2 is not sufficient and there is a low expression of all three genes, a myeloid progenitor state related to successful GATA switching and there is a high level of expression of PU.1, and a megakaryocyte/erythroid progenitor state related to successful GATA switching and there is a high level of expression of GATA-1.

A high order of multi-stability within HSCs is interesting because it fits with newer continuum [57] and diffusion map models [58] for hematopoiesis. In these models, HSCs can veer directly towards any of the lineage options that are available as a spectrum. Affiliation to a cell lineage occurs much earlier than previous thought, and the finding that supports this view is that HSCs are really a consortium of multipotent cells and subtypes that have an intrinsic bias/affiliation towards a cell lineage. The subtypes include megakaryocyte- [59], lymphoid-, myeloid- and dendritic cell-biased [60,61,62,63] HSCs and erythroid- and macrophage-affiliated HSCs [64].

**Figure 1:**
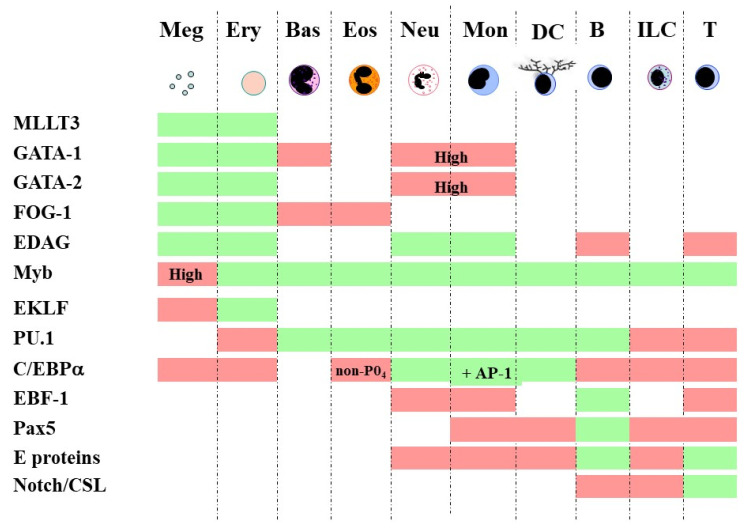
Shared usage of TFs by contiguous fates. The coordinated activity of multiple TFs is important to whether a cell adopts or avoids a particular fate. TFs can promote the development of one cell type or set of adjacent cell types and suppress neighboring fates. Boxes that are shaded green show that TFs must be active to direct the development of the particular cell type, and the TF inhibits the adoption of a fate for boxes that are shaded red (reviewed in [57]). Meg, megakaryocyte; Ery, erythroid; Bas, basophil/mast cell; Eos, eosinophil; Neu, neutrophil; Mon, monocyte; DC, dendritic cell; B, B cell; ILC, innate lymphoid cell; T, T cell.

Embedding bi-stable models together for multi-stability is in keeping with continuum and diffusion map models because developmental pathways are still placed close to one another. The adjacent relationships in a continuum model are megakaryocytes ↔ erythrocytes ↔ basophils/mast cells ↔ eosinophils ↔ neutrophils ↔ monocytes ↔ dendritic cells ↔ B cells ↔ innate lymphoid cells ↔ T cells [57]. They were inferred from close relationships between particular cell lineages as seen for HPCs when assayed in vitro. In addition, the differentiation of HSCs towards each of the various end cell types has long been attributed to a complex network of TFs. For fates that are contiguous in the above continuum, there is shared usage of transcription factors [57]. For example, GATA-1, GATA-2 and Friend of GATA-1 (FOG-1) are important for the megakaryocyte → erythrocyte → basophil/mast cell → eosinophil span of the spectrum [65], and GATA-1 restricts mast cell development [66], possibly by combining with FOG-1 to disrupt the association between GATA-1 and PU-1 [67]. In essence, TFs that play a role in lineage commitment set up a new gene expression pattern and extinguish others (Figure 1). The importance of the sharing of TFs to the level of gene expression noise within cells is considered below.

More recent molecular studies captured the global transcriptome of developing HSCs from single cell RNA sequencing and linked this information to cell fate. The near-neighbor pathways in a continuum were towards erythrocytes, basophils, neutrophils, monocytes, dendritic cells, B cells, and T cells [68], which is similar to the above. From the single cell RNA sequencing, HSC development is a progressive process with broad developmental trajectories allowing cells to move to the left or right of an initial chosen fate to one that is adjacent in the landscape [58]. Alternative fates might be viewed as remaining latent within HSCs and HPCs, and this plasticity is important to consideration of whether the progeny of CML LSCs are restricted to the neutrophil pathway because the cell-of-origin is an HSC with an intrinsic bias/affiliation towards neutrophils. This is unlikely because developing HSCs and HPCs can “change their mind”.

## 7. Noise and Bursting of Gene Expression

There remains an important question: How do lineage options arise within HSCs in the first instance? There is natural variation to the expression of lineage-affiliated genes within HSCs because, as mentioned above, sub-sets of mouse HSCs express mRNA for the receptor for Epo at a low level and the receptor for macrophage colony-stimulating factor (M-CSF) at their surface at a low level [64]. A much earlier finding from the use of RT-PCR to measure mRNA levels was that there was low level expression of a number of lineage-affiliated genes within the multipotent murine FDCP-mixA4 cells prior to cell lineage commitment. Variable expression was observed for single cells for the receptors for Epo, granulocyte colony-stimulating factor, granulocyte/macrophage colony-stimulating factor and M-CSF. Low level mRNA expression was promiscuous because β-globin (erythroid) and myeloperoxidase (myeloid) expression occurred within the same cell, and this might relate to transcriptional episodes and mRNA persistence or cells cycling through programs [69]. As to the noise within HSCs regarding the expression of genes that encode lineage-affiliated receptors, it is important to bear in mind that their cytokines can instruct HSC fate. M-CSF instructs myeloid lineage fate within single HSCs in vitro, and intravenous injection of recombinant M-CSF into mice increased activation of the myeloid-associated TF PU.1 in long term reconstituting-HSCs and the proportion of myeloid-biased HSCs [70]. Epo instructs an erythroid fate within multipotent HPCs and decreases myeloid output [71]. A low level of expression of cytokine receptors by HSCs cells may allow these cells to explore various fates leading to an eventual survival dependency as they differentiate further. For the soil bacterium *Pseudomonas putida*, it is interesting that noise within metabolic regulatory networks allows cells to explore various nutritional landscapes [72].

Noise is widespread within cells and is caused by the complex dynamics to the TF regulation of gene expression together with protein turnover. The gene cis-regulatory elements that are accessible to TFs, as described as chromatin nuclease hypersensitive sites, are scattered throughout the nucleus. TFs complexed with chromatin remodelers/modifiers and bound to cis-regulatory elements lead to the activation of gene expression. Gene expression noise is drastically enhanced by spatial fluctuations in TF levels, due to diffusion [73], and the sharing of TFs between genes [74]. As considered above, developing HSPCs share the usage of TFs to promote or prevent the adoption of a fate [57]. The strength of signals that a cell receives from its environment are integrated into the process of TF-mediated gene regulation and also influence noise.

**Figure 2:**
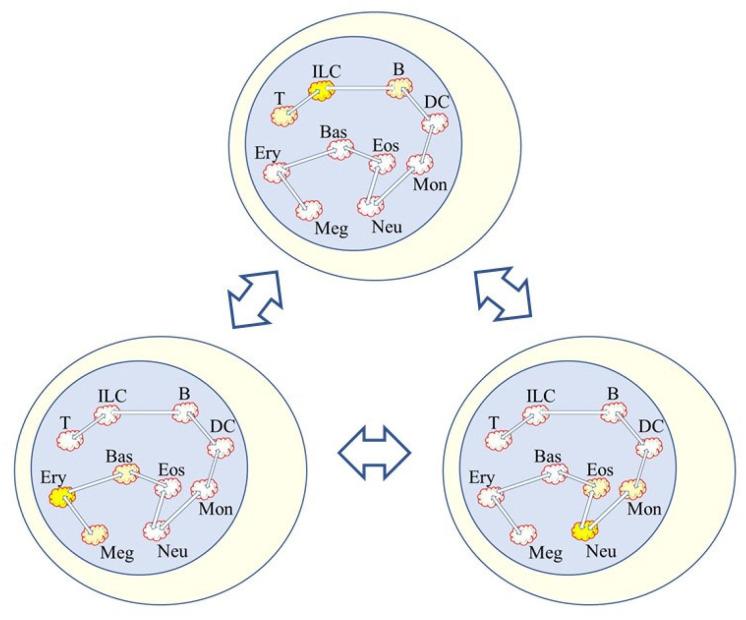
Low level bursting of gene expression within HSCs. HSCs are able to ‘choose’ directly from all of the options. The bursting of gene expression at a various site for a particular fate is shown by the bright yellow highlighted clouds. The noise is not exclusive to just a pathway of development because developmental trajectories are broad and there are near-neighbor relationships between the cell lineages. The close relationships shown between the pathways of development are as for continuum and diffusion map models of hematopoiesis. Trajectories are broad and binary flips to an adjacent landscape are shown by the clouds with a reduced yellow highlighting. The depiction is in keeping with the mathematical embedding together of bi-stable models.

Hence, noise and the bursting of gene expression at various sites throughout the genome is very likely to play a role in how lineage options are made available to HSCs (Figure 2). It has been proposed that noise distorts the epigenetic landscape to shape cell decision-making [75]. Furthermore, to take-into-account that the signals received by a cell influence noise to distort the epigenome, its geometric landscape has been modelled for ES cells directed by appropriate signals towards the neural and mesoderm fates [76]. However, and by contrast to a focus on changes to the epigenetic landscape guiding decision-making, the promyeloid cell line HL60 is able to differentiate into macrophages, neutrophils, monocytes, and monocyte-derived macrophages, and when HL60 cells differentiated along these pathways there were few differential changes in the chromatin landscape for up to 24 h. Instead, changes occurred during the middle to late stages of differentiation [77].

## 8. Waking up Old and Damaged Cells

As mentioned above, the *BCR-ABLp210* oncogene occurs in the cells of individuals who do not develop CML, and these cells are apparently normal. An entirely new view of cancer has questioned whether oncogenes are the tipping point to the onset of cancer. Of interest were why non-smokers get lung cancer, if carcinogen-induced DNA damage is all important, and how air pollutant particulate matter 2.5 (PPM2.5) causes cancer without damaging DNA. As to the latter and for mice, PPM2.5 exposure led to the release of interleukin-1β in the lung, which caused inflammation, whereby activated cells help to repair lung damage. Blocking the action of interleukin-1β and inflammation prevented the formation of lung cancers. Additionally, a surprising finding was that the risk of lung cancer was cut when the action of interleukin-1β was blocked in a cardiovascular disease trial. Thus, how might the release of interleukin-1β be the tipping point to lung cancer? For a 50-year old person, lung cells with potentially cancer mutations are at a frequency of around one in every 600,000 cells, due to damage to our cell’s DNA as we age. The new postulate is that the released interleukin-1β had woken up damaged cells that appear to be healthy but are normally inactive to give rise to lung cancer [78]. 

**Figure 3:**
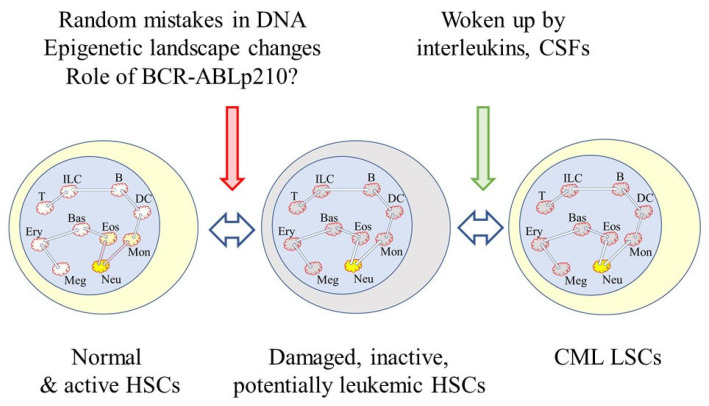
Waking up of damaged cells that pre-exist. Damage to the normal HSC genome occurs from random mistakes during DNA replication, and BCR-ABLp210 is known to enhance DNA damage. The BCR-ABLp210 tyrosine kinase facilitates the repair of DNA double-strand breaks, but it has been proposed that there is genetic instability within CML cells due to the unfaithful repair of double stranded breaks. There are BCR-ABLp210-provoked changes to the epigenetic landscape. The damaged cells are apparently normal but potentially cancerous. They are inactive and woken up by signals received from the environment, perhaps from interleukins or colony stimulating factors (CSFs).

The waking up of pre-existing and damaged cells that appear to be normal may be an element that is missing from our understanding of the onset of CML. There is damage to the genome of stem cells as they divide to replenish themselves. Three mutations occur every time they divide, resulting from random mistakes during DNA replication [79]. For self-renewing tissues, more than half of the somatic mutations occur prior to the initiation of tumors [80]. There is also a strong correlation between normal stem cell divisions and cancer incidence as seen from studies of the risk of 17 types of cancer [81]. The lineage capabilities of HSCs changes with age because there is a predominance of myeloid-biased cells within the HSC compartment of aged mice [61,82]. The complex process of retention of the availability of all of the lineage options might, therefore, be prone to error. As to an increase in myeloid-biased of HSCs with age, it is interesting to note that around 50% of CML patients are aged 66 and older [83]. The new view to the onset of lung cancer may apply to many, if not all, cancers. Therefore, an as-yet-unseen interleukin or colony-stimulating factor may play a role in the overproduction of neutrophils by CML LSCs as a counterpart to age-related damage to the genome, *BCR-ABLp210* expression and changes to the epigenetic landscape (Figure 3).

## 9. Concluding Remarks

The behavior of CML LSCs is very different from that of HSCs. There is substantial natural variation to lineage options within HSCs, and for CML LSCs there is an intrinsic stability regarding the neutrophil fate during the chronic phase of disease. Global changes to the epigenome are important, as seen from the studies of transgenic mouse models of CML, and they may influence the noise to/priming of lineage fates and bursting of gene expression. CML might then be viewed as a perturbation to the epigenetic landscape to normal stem cell development. During HSC cell fate specification there is likely to be the need to buffer noise to ensure a ‘chosen’ outcome. For CML LSCs, we might speculate that there is inappropriate/excessive buffering of fate options and that myeloid fate is facilitated by BCR-ABLp210 or some other event. From the transgenic mouse studies and as seen for the progeny of BCR-ABLp210+ hemangioblasts, BCR-ABLp210 can veer HSC development towards a myeloid fate. For BCR-ABLp210-mediated transformation, there may be a connection between BCR-ABLp210 expression and the bursting of myeloid gene expression at this moment in time. The waking up of apparently healthy, inactive, and damaged cells adds a further consideration to the onset and nature of CML, but the importance of this new view on cancer to CML remains to be seen.

Presently, we do not have a clear explanation as to why the progeny of CML LSCs are directed towards a neutrophil fate during the chronic phase. The way forward is to develop a deeper understanding of how complex networks of cis-regulator elements and TFs and changes to the epigenetic landscape cooperate to allow HSCs to affiliate to a cell lineage. It seems that the epigenetic landscape is the judge, jury and executioner, but the precise manner of this entire process is still unclear. Even so, perhaps CML disease and other tissue-specific stem cell cancers arise from deregulation of the epigenome.

## Data Availability

Not applicable.
